# Case Report: Massive Intestinal Pneumatosis and Pneumoretroperitoneum Following Hematopoietic Stem Cell Transplantation in a 2-Year-Old Child

**DOI:** 10.3389/fped.2021.700736

**Published:** 2021-12-08

**Authors:** Giorgia Contini, Arianna Bertocchini, Roberto Carta, Pietro Merli, Alessandro Inserra, Pietro Bagolan, Francesco Morini

**Affiliations:** ^1^Medical and Surgical Department of the Fetus, Neonate and Infant, Bambino Gesù Children's Hospital, IRCCS, Rome, Italy; ^2^Department of Pediatric Surgery, Bambino Gesù Children's Hospital, IRCCS, Rome, Italy; ^3^Department of Pediatric Hematology and Oncology, Bambino Gesù Children's Hospital, IRCCS, Rome, Italy

**Keywords:** hematopoietic stem cell transplantation, intestinal pneumatosis, graft versus host disease (GVHD), pneumomediastinum, severe combined immunodeficiency

## Abstract

A 2-year-old boy with severe combined immunodeficiency (SCID) developed intestinal graft-versus-host disease (GVHD) after hematopoietic stem cell transplantation (HSCT), associated with massive intestinal pneumatosis (IP), pneumoretroperitoneum (PRP), and pneumomediastinum. His fair clinical conditions allowed conservative management, with progressive normalization of imaging findings. The patient did not require surgery and is alive and in good clinical conditions at follow-up. In children with GVHD-related IP but good clinical conditions and no signs of peritonitis, IP is not a mandatory indication for surgery, despite its potentially striking imaging features. Conservative management, with intestinal rest, decompression, and antibiotics, often allows regression of the clinical picture.

## Introduction

Graft-versus-host disease (GVHD) is the most common immunological complication of allogeneic hematopoietic stem cell transplantation (HSCT); alloreactive donor T cells recognize recipient's cells as non-self, causing direct and indirect damages to different organs. The most affected organs are the skin, the liver, and the gastrointestinal tract (GI) (1).

GVHD can be classified, based on type of signs/symptoms, time from HSCT, and immunological feature, as acute or chronic; moreover, overlap forms rarely occur. Acute GVHD (aGVHD) usually develops within the first 100 days of HSCT and can involve the skin, liver, and GI. Chronic GVHD (cGVHD) can occur *de novo* or following aGVHD, arising several months after HSCT, and can involve almost any organ. This complication leads to significant morbidity, reduced quality of life, and decreased overall survival. In children the rate of cGVHD tends to be lower (20–50%) than in adults (60–70%) (2) and most commonly involves the skin, eyes, oral cavity, GI, liver, and lungs.

Rarely, GVHD following HSCT presents with intestinal pneumatosis (IP) (3, 4). IP is usually an indication for surgery. However, it is important to understand when to pursue conservative management of IP, especially in children with complex medical problems, such as GVHD, also to avoid unnecessary and potentially harmful surgery (5).

We present the case of a child with intestinal cGVHD following intestinal aGVHD after bone marrow transplantation (BMT) who developed massive IP with pneumoretroperitoneum (PRP) but no signs of pneumoperitoneum (PP) or peritonitis.

## Case Description

A 2-year-old boy was referred to our hospital for the suspect of gut GVHD following HSCT for severe combined immunodeficiency (SCID), with T-B+NK- phenotype, due to IL2RG mutation (c.C375A:p.Y125^*^). No pathological conditions were recognized in the patient's family; personal medical history was positive for weight loss associated with frequent airway infections.

Three months before referral to our hospital, he underwent HSCT from an unrelated cord blood unit (2 × 10^5^ CD34+/kg; compatibility 7/8 for an allelic mismatch in locus A; blood groups: donor 0 positive, recipient A negative).

Reduced intensity conditioning (RIC) included fludarabine (30 mg/m^2^/day for 5 days) and treosulfan (12 g/m^2^/day from day −7 to day −5). Anti-thymocyte globulin (ATG), at a dose of 2.5 mg/kg/day, was employed from day −10 to day −8). GVHD prophylaxis therapy consisted of ciclosporin intravenously (2 mg/kg/day) and prednisone (1 mg/kg/day) until day +30. After 1 month from the HSCT, he developed acute grade II skin GVHD localized to the head and trunk, which required an increase in steroid therapy, and 3 months after transplantation, he developed diarrhea with negative stool culture and negative research for *Clostridium difficile*. Intestinal GVHD was suspected, and he was referred to our hospital for further management.

A colonoscopy and an esophagogastroduodenoscopy with biopsies were performed confirming the diagnosis of intestinal GVHD (grade 2 according to Lerner scale). At the same time, a bone marrow aspirate was performed, confirming mixed chimerism. Methylprednisolone at 2 mg/kg was started. In the following weeks there was an improvement of donor chimerism but a worsening of the intestinal GVHD that progressed to grade IV (bloody diarrhea), despite treatment intensification with begelomab and mycophenolate mofetil. Ustekinumab was also added because of non-response. Additionally, ruxolitinib was also initiated with partial response. In the following months, the patient suffered from several episodes of intestinal sub-occlusion alternating with bloody diarrhea associated with septic episodes due to bacterial translocation. Transplantation of intestinal microbiota was also attempted without significant clinical response.

**Figure 1:**
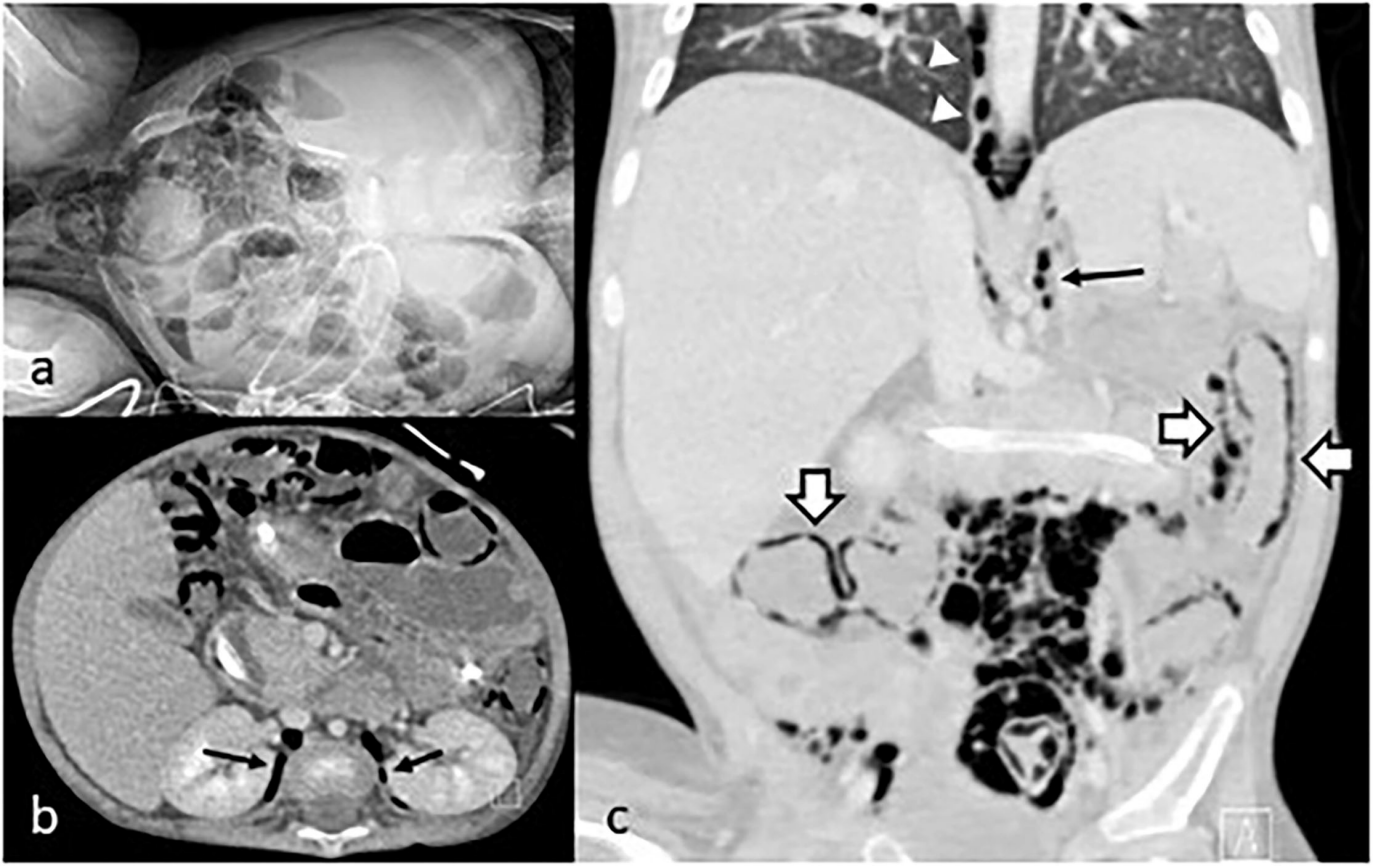
**(a)** Abdominal x-rays showing IP; **(b)** coronal CT scan with PRP (arrows); **(c)** sagittal CT scan showing pneumomediastinum (arrowhead), PRP (arrow), and IP (empty arrows).

Eight months after the diagnosis of intestinal GVHD the patient developed acute pancreatitis and severe abdominal pain. Blood tests showed C-reactive protein (CRP) 11.69 mg/dl (normal < 0.5 mg/dl), procalcitonin (PCT) 1.54 ng/ml (normal < 0.5 ng/ml), lipase 1,800 IU/L (normal <160 IU/L). Abdominal x-rays and computed tomography (CT) scan showed marked bowel loops distention, massive IP, PRP, and pneumomediastinum (Figure 1). Despite the radiological findings, his clinical conditions were fair, with abdominal pain and distention but no signs of peritonitis. In view of his fair clinical conditions and laparotomy-related risks, conservative management was carried out, with intestinal rest, decompression with nasogastric tube placement, broad-spectrum antibiotics, and tight clinical observation (e.g., abdominal x-rays every 24 h).

**Figure 2:**
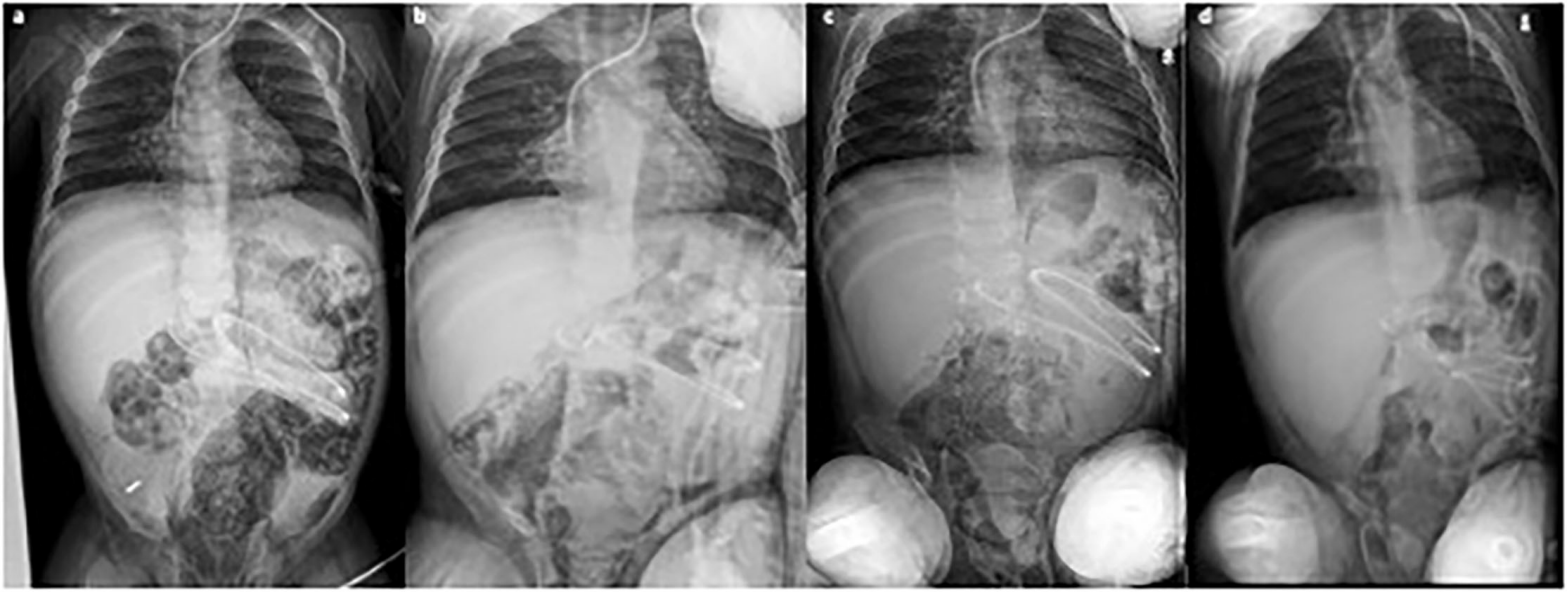
Consecutive abdominal x-rays [**(a)** day 1; **(b)** day 3; **(c)** day 5; **(d)** day 11] showing progressive reduction of intestinal pneumatosis. Clinical conditions remained fair during the whole period.

**Figure 3:**
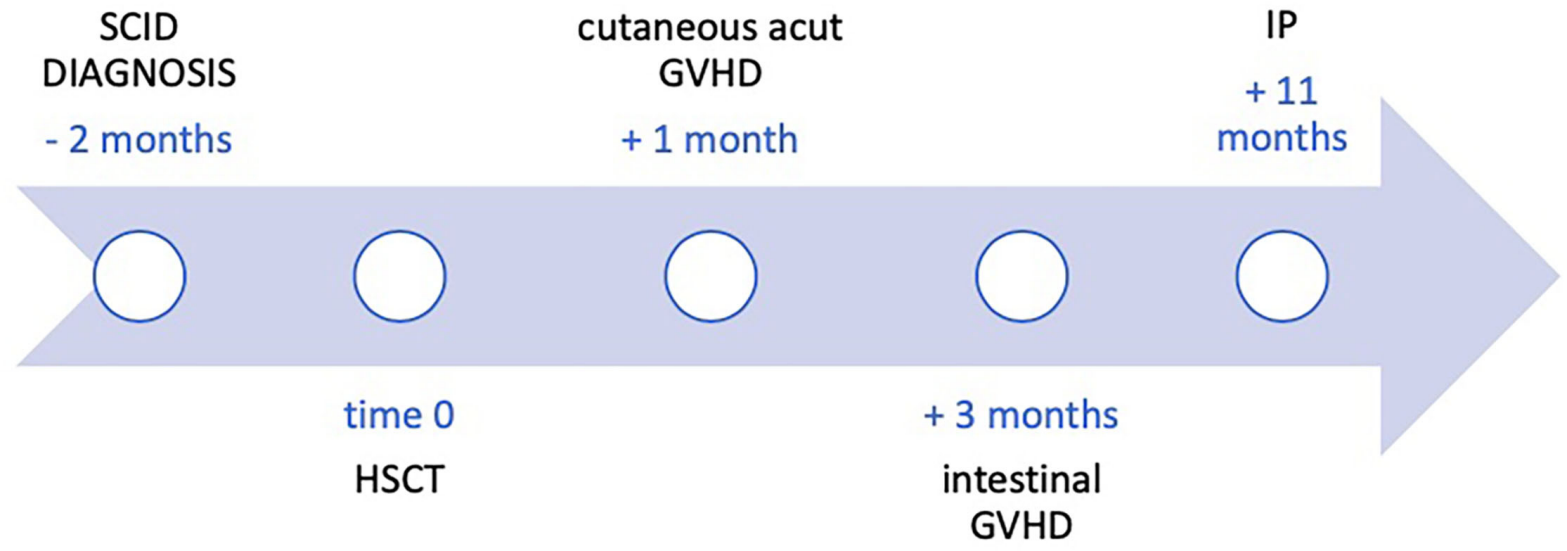
Timeline with relevant data from the episode of care.

After an initial increase of both IP and PRP, imaging findings progressively returned to normal within 12 days (Figure 2). The patient did not require abdominal surgery and is alive at 6 months of follow-up; however, despite several lines of immunosuppressive therapy, he still presents recurrent flare of GI GVHD (Figure 3).

## Discussion

We report on a child with SCID who developed IP, PRP, and pneumomediastinum associated with GVHD following HSCT.

IP is defined as the presence of gas within the bowel wall. In some cases, gas may be also observed in the intraperitoneal and extraperitoneal spaces and sometimes is accompanied by a local and systemic inflammatory reaction. It is not a disease *per se* but a radiologic and pathologic finding that can be associated with a variety of disorders, including chronic obstructive pulmonary disease, gastrointestinal obstruction, bowel ischemia, neonatal necrotizing enterocolitis, immunodeficiency syndromes, and bacterial or viral infections; moreover, it can occur after surgical procedures as well as colonoscopies (6). IP has also been reported in individuals with acquired immunodeficient states and in those who have undergone BMT, as a manifestation of GVHD (7). Gut-associated lymphoid tissue is the most extensive lymphoid organ in the body. In primary immunodeficiency disorders, dysregulation of immunity can occur in a wide array of gastrointestinal diseases. In particular, SCID patients are prone to gastrointestinal manifestations of GVHD after blood transfusions or an allogenic BMT (8). The pathogenesis of GVHD after HSCT is multi-factorial. Several elements may be associated with the development of IP, including pre-transplantation chemotherapy and radiotherapy, steroid therapy, infectious colitis, and septic shock (9). Intestinal GVHD leads to atrophic mucositis with ulcer formation, bacterial and fungal superinfection, fibrosis, and development of malabsorption syndromes. Damage to the intestinal mucosa, coexisting infection, and inflammatory infiltration with concomitant steroid therapy may predispose to IP. Lymphocyte depletion induced by prolonged steroid therapy causes an alteration of lymphoid follicles. These anatomic and functional defects, associated with the inflammatory process, can produce damage to muscolaris mucosae and allow the passage of intraluminal bacteria or gas into the submucosa (10).

In our patient, both the episodes of intestinal infection with bacterial translocation and imbalance of GI microbiota and prolonged immunosuppressive and steroidal therapy may have played an important role in the pathogenesis of IP.

IP may be associated with a heterogeneous clinical picture. In some cases it is asymptomatic, but in most patients, it presents with abdominal pain, nausea, vomiting, and diarrhea (11, 12).

Diagnosis of IP usually comes from an abdominal x-ray. In selected patients, the addition of abdominal CT scan may allow the identification of further signs reflecting the severity of the pathology, such as intestinal wall thickening, other gas collection (such as PRP in our patient), pathological bowel wall or soft tissues contrast enhancement, bowel lumen dilatation, fluid in the peritoneal cavity, and gas in the portal vein (13).

In children abdominal ultrasound can also detect IP and other important signs such as bowel distension, bowel wall thickness, portal venous gas, and free abdominal air as an abdominal x-ray. Moreover, abdominal ultrasound can show free and focal fluid collections, the status of peristalsis, and the presence or absence of bowel wall perfusion using Doppler imaging, with the advantage of the absence of ionizing radiation. However, the results of the latter technique are very operator-dependent (14, 15).

In our patient, IP was associated with PRP and pneumomediastinum but not PP nor clinical manifestations of peritonitis such as worsening of general conditions, abdominal tenderness, and abdominal wall discoloration. The presentation suggested a confined condition that allowed us to undertake conservative management. Generally, in IP without PP, conservative management, including intravenous antibiotics and bowel rest for at least 7 days, should be the first-line treatment. This is especially true in complex patients such as those with intestinal GVHD, where surgical stress may be particularly harmful. Enteral feeding may be gradually re-introduced when laboratory and imaging findings suggest regression of the infectious/inflammatory process. Parenteral nutrition is administered until feeding is fully re-established (3). Also, in adults with IP (but no signs of peritonitis) following BMT, conservative management has been recommended (4).

Conversely, in most cases of IP with associated PP, emergency surgery is mandatory. Although PP associated with IP without peritonitis has been rarely reported in the literature (3, 16), the presence of PP is often synonymous with intestinal perforation and peritonitis, which in turn may be the motor for systemic inflammatory reaction syndrome and multiple organ dysfunction.

To complicate the picture further, in patients with IP and PP following HSCT, this treatment paradigm should be taken with caution as PP is not always a synonym for intestinal perforation. Korhonen and coworkers analyzed the incidence and outcome of IP among 178 children who underwent allogenic HSCT between September 1999 and February 2010. Eighteen of 178 patients (10.1%) developed IP at a median of 94 days (range, 11–1,169 days) after transplant. All patients presented with either abdominal pain or distention, and half of the patients had free air on radiographs but no obvious manifestations of acute peritonitis. All patients were managed conservatively without surgery. Transplant-related mortality was significantly higher in patients who developed IP compared to those who did not, but no deaths were directly attributable to IP (17).

In conclusion, in children with GVHD, IP is a rare complication that may represent a conundrum for treating physicians and surgeons. Imaging findings alone may depict a situation that seems more severe than it really is. If clinical conditions are stable, treatment should be as conservative as possible. In these complex patients, surgical exploration should be reserved for those with clinical symptoms of peritonitis, signs of intestinal perforation, or obstruction.

## Data Availability Statement

The original contributions presented in the study are included in the article/supplementary material, further inquiries can be directed to the corresponding author.

## Ethics Statement

Written informed consent was obtained from the parents of the patient for the publication of any potentially identifiable images or data included in this article.

## Author Contributions

GC, FM, AB, RC, and PM contributed to conception and design of the article. GC wrote the manuscript. FM wrote sections of the manuscript. All authors contributed to manuscript revision, read, and approved the submitted version.

## Conflict of Interest

The authors declare that the research was conducted in the absence of any commercial or financial relationships that could be construed as a potential conflict of interest.

## Publisher's Note

All claims expressed in this article are solely those of the authors and do not necessarily represent those of their affiliated organizations, or those of the publisher, the editors and the reviewers. Any product that may be evaluated in this article, or claim that may be made by its manufacturer, is not guaranteed or endorsed by the publisher.

## Abbreviations

aGVHD: acute graft-versus-host disease
cGVHD: cronic graft-versus-host disease
CT: computed tomography
GVHD: graft-versus-host disease
HSCT: hematopoietic stem cell transplantation
IP: intestinal pneumatosis
PRP: pneumoretroperitoneum
SCID: severe combined immunodeficiency
BMT: bone marrow transplantation.
